# Low drying temperature has negligible impact but defatting increases in vitro rumen digestibility of insect meals, with minor changes on fatty acid biohydrogenation

**DOI:** 10.1186/s40104-025-01199-5

**Published:** 2025-05-07

**Authors:** Manuela Renna, Mauro Coppa, Carola Lussiana, Aline Le Morvan, Laura Gasco, Lara Rastello, Jonas Claeys, Gaëlle Maxin

**Affiliations:** 1 Department of Veterinary Sciences, University of Turin, Largo P. Braccini 2, 10095 Grugliasco, TO Italy; 2 Department of Agricultural, Forest and Food Sciences, University of Turin, Largo P. Braccini 2, 10095 Grugliasco, TO Italy; 3 UMR 1213 Herbivores, Université Clermont Auvergne, INRAE, VetAgro Sup, 63 122 Saint-Genès-Champanelle, France; 4 Insect Research Centre, Inagro, Ieperseweg 87, 8800 Rumbeke-Beitem, Belgium

**Keywords:** Ammonia, Black soldier fly, Defatted insect meal, Ether extract, Fatty acid, Full-fat insect meal, In vitro rumen fermentation, Methane, Yellow mealworm

## Abstract

**Background:**

Insect meals have been identified as innovative and sustainable feedstuffs that could be used in ruminant nutrition. However, current research on the effects that their processing may have on rumen digestibility and fatty acid (FA) biohydrogenation is scant. This trial aims to investigate the effects (i) of drying temperature of full-fat *Hermetia illucens* (HI) and *Tenebrio molitor* (TM) meals, and (ii) of residual ether extract (EE) content of defatted HI and TM meals, on their fermentation characteristics and FA of rumen digesta after 24-h in vitro rumen incubation.

**Methods:**

The tested full-fat meals included four HI and four TM meals obtained applying drying temperatures ranging from 30 °C to 70 °C, while the tested defatted meals consisted of five HI and two TM meals containing a residual EE content ranging from 4.7 to 19.7 g EE/100 g dry matter (DM). The applied statistical models (GLM ANOVA) tested the effects of insect species, drying temperature (full-fat meals) or EE content (defatted meals), and their interaction.

**Results:**

Drying temperature had minor effects on in vitro ruminal digestibility and FA profile of rumen digesta. Irrespective of insect species, increasing the drying temperature led to a reduction of in vitro degradation of proteins from insect meals, as outlined by the significant decrease in ammonia production (−0.009 mmol/g DM and −0.126 g/100 g total N for each additional 1 °C). Irrespective of insect species, defatting increased total gas, volatile fatty acids (VFA) and CH_4_ productions, and the proportions of total saturated and branched-chain FA in rumen digesta (+0.038 mmol/g DM, +0.063 mmol/g DM, +12.9 µmol/g DM, +0.18 g/100 g FA, and +0.19 g/100 g FA for each reduced 1 g EE/100 g DM, respectively), and reduced the proportion of total PUFA (−0.12 g/100 g FA).

**Conclusions:**

The applied drying temperatures of full-fat insect meals are too low to exert impactful effects on rumen digestibility and FA biohydrogenation. Fat lowered fermentation activity, probably because of an inhibitory effect on rumen microbiota. The increased ruminal digestibility of defatted insect meals suggests that they can be more suitable to be used in ruminant nutrition than full-fat ones.

**Supplementary Information:**

The online version contains supplementary material available at 10.1186/s40104-025-01199-5.

## Background

In the last decades, concerns on global sustainability have increased year by year. Hence, current ruminant nutrition research is focusing on reducing the environmental impact of farming activities and promoting the application of circular economy models [1].

Insect rearing contribute to circular economy by efficiently managing organic waste, revalorizing it to produce nutrients that can be used to feed both humans and animals [2]. Insect-derived products (meals and oils) are considered suitable alternatives to conventional ingredients as, when used in feed formulations, they can help reducing the environmental impact of the livestock sector [3, 4]. Insect-derived products are characterized by valuable nutritional compositions, being rich in proteins with good amino acid profiles, rich in lipids with highly variable fatty acid (FA) profiles (depending on insect species, rearing substrate and processing methodologies), but also containing appropriate vitamin (including vitamin B_12_ that lacks in plant protein sources) and mineral contents [5]. Among the edible insects that have been tested for feed purposes so far, *Hermetia illucens* (HI) and *Tenebrio molitor* (TM) are the most studied due to their rapid growth, easy management and, especially for HI, the possibility to be reared on a wide range of organic substrates [6, 7].

At the end of the rearing period, insect larvae can be delivered live to poultry [8, 9] or can be transformed into meals and oils to be included in compound feed [10]. For full-fat meals preparation, post killing phases contemplate drying procedures with the aim of reducing water and water activity to 5%–10% and < 0.60, respectively, thus avoiding microbiological contamination and spoilage [11]. Temperature-time levels and combinations may vary based on insect species, applied killing procedures and drying method (e.g., oven, microwave) used [12]. Several authors compared the effects of different drying methods on the quality of insect meals [13, 14]. However, the potential effects of drying temperatures on the physico-chemical and sensorial attributes of insect meals still need to be thoroughly studied and understood [15, 16].

Because of their high fat content, the dietary inclusion of full-fat insect meals would be prone to determine an excess of dietary lipid supply [17] and could lead to pellet stability issues [18]. Thus, most industries are applying defatting technologies to obtain more stable insect meals, characterized by lower ether extract (EE) and, at the same time, higher crude protein (CP) concentrations when compared to full-fat meals [19]. The defatting process also results in an increase of the shelf-life of the meals [20]. In any case, defatting processes are not standardized yet, leading to a variability in fat content ranging from about 5% to 20% EE [21, 22].

When focusing on ruminant nutrition, heat treatments of protein sources can decrease CP and dry matter (DM) degradability at ruminal level [23], thus affecting the animal performance. Moreover, so far research on the use of insects as feed has mainly focused on full-fat meals [24]. While studying the in vitro rumen fermentation characteristics and lipid biohydrogenation of full-fat meals from eight different insect species, Renna et al. [25] found lower total gas production, CH_4_ production, volatile FA production, and in vitro organic matter disappearance when compared to plant meals conventionally used in ruminant nutrition. Defatting could be a solution to enhance digestibility of insect meals for ruminants [24], and the effect of the residual EE of defatted insect meals on ruminant nutrition needs to be investigated.

In this context, this trial aims to investigate, in vitro, the effects that insect processing [i.e., (1) drying temperature of full-fat HI and TM meals, and (2) residual EE content of defatted HI and TM meals] has on rumen fermentation characteristics and FA profile of rumen digesta.

## Methods

This trial received authorization from the French Ministry for Research (No. 7138-2016092709177605v6). Care and use of live animals employed in this research followed the European Union Directive 2010/63/EU, reviewed by the French local ethics committee (C2E2A, “Comité d’Ethique pour l’Expérimentation Animale en Auvergne”).

### Insect meals

Fifteen insect meals were used in this study. In detail, eight full-fat insect meals were obtained starting from HI and TM populations that have been bred at the Insect Research Centre of Inagro (Roeselare-Beitem, Belgium) since 2013. The HI and TM larvae were killed by deep freezing (−20 °C) and then dried at different temperatures (Vötsch VTU 100/150; Weiss Technik, Reiskirchen, Germany), as follows: four HI meals oven-dried to a constant weight at 40 °C (HI 40°C), 50 °C (HI 50 °C), 60 °C (HI 60 °C), 70 °C (HI 70 °C), and four TM meals at 30 °C (TM 30 °C), 50 °C (TM 50 °C), 60 °C (TM 60 °C), 70 °C (TM 70 °C). The lowest drying temperature is different between HI and TM meals because we encountered some technical problems with the oven during the drying of TM meal at 40 °C, so this batch could only be exposed to 30 °C. The other seven insect meals were instead provided by different European suppliers and submitted to defatting processes, resulting in different residual EE contents [five HI meals containing 19.7 (HI 19.7), 12.8 (HI 12.8), 9.2 (HI 9.2), 7.0 (HI 7.0), and 4.7 (HI 4.7) g EE/100 g DM, and two TM meals containing 8.1 (TM 8.1) and 5.7 (TM 5.7) g EE/100 g DM]. All the HI and TM used to produce the defatted meals were raised on vegetable substrates and the meals were obtained from their larval stage. Further information on the technological processes applied to obtain the defatted insect meals, being covered by intellectual property rights, was not provided by the suppliers.

### In vitro fermentation

Fermentations were performed using a batch technique. The donor animals were four Texel adult castrated male sheep fitted with a rumen cannula and weighing, on average, 64.6 ± 7.10 kg. The animals were individually fed daily hay from permanent grassland (840 g DM) and concentrate (360 g DM), the meal being divided into equal amounts at 08:30 and 16:00 h. The sheep were adapted to the diet for 15 d before being used as donors.

Two sets of incubations were performed. The first set was composed by the full-fat meals obtained applying different drying temperatures. The second set was composed by the defatted meals and was run simultaneously to that described by Renna et al. [25]. For both incubation sets, four series of 24 h incubations (statistical replications) were performed, one per animal. In each series, the insect meal samples were anaerobically incubated in duplicate, using the rumen fluid from one animal, as detailed by Renna et al. [25]. Briefly, a sample of the solid fraction of the ruminal content was obtained through the cannula before the morning feeding and squeezed through a polyester monofilament cloth. Rumen fluid was diluted in anaerobic phosphate:carbonate buffer solution, as described by Theodoridou et al. [26]. Forty mL of this buffered rumen solution were added in pre-warmed (39 °C) and N_2_-flushed 120-mL serum bottles containing 600 ± 0.5 mg DM of insect meal sample (two bottles per meal sample; i.e., technical replications). The bottles were sealed hermetically to ensure anaerobic conditions and immediately manually shaken. Then, they were placed in a water bath at 39 °C for 24 h. Four additional bottles containing only buffered rumen fluid (blanks) were incubated simultaneously. The bottles were manually shaken 1 h 30 min, 3 h, 5 h, 7 h and 23 h 30 min after the beginning of the fermentation, and the fermentation was stopped after 24 h. Gas production was measured at 24 h using a pressure transducer [27]. At 24 h, after recording pressure, a gas sample was taken for gas composition analysis, and pH was measured.

Rumen digesta was then centrifuged at 3,000 × *g* for 10 min at 4 °C. For volatile fatty acids (VFA) determination, 0.8 mL of the supernatant were mixed with 0.5 mL of 4 mg/mL crotonic acid and 20 mg/mL metaphosphoric acid in 0.5 mol/L HCl. The mixture was cooled at 4 °C for 2 h, centrifuged at 16,500 × *g* for 10 min at 4 °C, and the resulting supernatant was frozen at −20 °C for VFA analysis. For ammonia determination, 1.6 mL of the supernatant was transferred to a polypropylene tube containing 0.16 mL of H_3_PO_4_ 5% (v/v) and frozen at −20 °C until analysis.

Four additional series of incubations were performed to determine the FA composition of the non-degraded particle residues, as detailed by Renna et al. [25]. The residues were freeze-dried (MUT PCCPLS1.5 001 A, Cryotec, France) for FA determination.

### Laboratory analysis

#### Chemical composition of the insect meals

After being ground using a knife mill (Grindomix GM200, Retsch GmbH, Haan, Germany; final fineness < 300 μm), the meals were analysed for their chemical composition and FA profile. The DM, ash, CP, acid detergent fibre (ADF) and acid detergent lignin (ADL) contents were assessed by means of AOAC procedures (methods No. 930.15, 942.05, 984.13 and 973.18, respectively) [28]. The nitrogen-to-protein conversion factors (Kp) of 4.67, 4.75, 5.62, and 5.59 were used to calculate the CP contents of full-fat HI meals, full-fat TM meals, defatted HI meals, and defatted TM meals, respectively [29]. Method No. 2003.05 of the AOAC International [30] was used to assess the EE content of the meals. The neutral detergent fibre (NDF) content of the meals was determined adding α-amylase (Merck, Darmstadt, Germany) and sodium sulphite (Merck, Darmstadt, Germany) and correcting the results for residual ash content [31]. The neutral-detergent and acid-detergent insoluble nitrogen (NDIN and ADIN, respectively) were determined following the procedures described in Licitra et al. [32]. The chitin content of the meals was analysed according to Woods et al. [33], with the modifications detailed by Gasco et al. [34]. The chemical composition of the meals, expressed as g/100 g DM, is reported in Table 1.

**Table 1:** Chemical composition of the insect meals, g/100 g DM (unless otherwise stated)

**Item**	**Full-fat meals obtained with different drying temperature**^**a**^								**Defatted meals with different residual ether extract content**^**b**^						
	**HI 40 °C**	**HI 50 °C**	**HI 60 °C**	**HI 70 °C**	**TM 30 °C**	**TM 50 °C**	**TM 60 °C**	**TM 70 °C**	**HI 19.7**	**HI 12.8**	**HI 9.2**	**HI 7.0**	**HI 4.7**	**TM 8.1**	**TM 5.7**
DM, g/100g	89.1	89.8	93.1	91.8	92.9	94.3	95.6	96.2	95.0	93.4	93.6	94.4	94.0	93.4	92.5
OM	88.7	88.6	88.7	88.7	94.2	94.5	94.5	94.7	93.3	89.8	88.2	87.8	91.8	94.7	94.8
CP	35.7	35.6	35.3	35.9	44.7	45.0	45.0	45.5	41.2	50.9	51.6	50.3	61.3	56.5	63.8
EE	24.0	23.8	24.2	23.9	26.0	25.7	25.6	25.3	19.7	12.8	9.2	7.0	4.7	8.1	5.7
NDF	28.9	28.3	28.4	28.9	23.4	23.6	23.1	23.6	31.5	25.7	26.8	29.8	25.4	22.5	24.8
ADF	6.3	6.6	6.5	6.0	4.0	3.9	3.1	3.8	8.3	8.8	9.1	14.2	10.1	8.3	9.1
ADL	1.5	1.6	1.5	1.5	1.1	1.0	1.0	1.1	0.4	0.8	1.1	1.9	1.2	1.6	0.6
NDIN	2.8	3.0	2.9	2.8	2.5	2.4	2.3	2.4	4.0	3.9	4.1	3.8	3.2	2.4	4.2
ADIN	2.5	2.6	2.5	2.5	1.9	1.8	1.8	1.9	3.2	3.1	3.1	3.0	2.6	2.0	3.3
Chitin	4.5	5.2	4.3	5.0	6.3	6.5	6.4	6.6	5.5	6.8	7.0	7.4	8.4	6.3	7.8

*DM* Dry matter, *HI Hermetia illucens*, *TM Tenebrio molitor*, *OM* Organic matter, *CP* Crude protein, *EE* Ether extract, *NDF* Neutral detergent fibre, *ADF* Acid detergent fibre, *ADL* Acid detergent lignin, *NDIN* Neutral detergent insoluble nitrogen, *ADIN* Acid detergent insoluble nitrogen

^a^Full-fat insect meals obtained applying different drying temperatures: HI meals processed at 40 °C (HI 40 °C), 50 °C (HI 50 °C), 60 °C (HI 60 °C) and 70 °C (HI 70 °C); TM meals processed at 30 °C (TM 30 °C), 50°C (TM 50 °C), 60 °C (TM 60 °C) and 70 °C (TM 70 °C).

^b^Defatted insect meals containing different residual ether extract (EE) content: HI meals containing 19.7 (HI 19.7), 12.8 (HI 12.8), 9.2 (HI 9.2), 7.0 (HI 7.0) and 4.7 (HI 4.7) g EE/100 g DM; TM meals containing 8.1 (TM 8.1) and 5.7 (TM 5.7) g EE/100 g DM

The meals were analysed for their FA profile using a combined direct transesterification and solid-phase extraction method, as reported by Renna et al. [35]. Separation, identification and quantification of FA methyl esters were performed as detailed by Renna et al. [36]. The results of the FA profile of the meals was expressed as g/100 g total FA, while the total FA content of the meals was expressed as g/kg DM. The FA profile of the meals is reported in Table 2.

**Table 2:** Fatty acid profile of the insect meals, g/100 g total FA (unless otherwise stated)

**Item**	**Full-fat meals obtained with different drying temperature**^**a**^								**Defatted meals with different residual ether extract content**^**b**^						
	**HI** **40 °C**	**HI** **50 °C**	**HI** **60 °C**	**HI** **70 °C**	**TM** **30 °C**	**TM** **50 °C**	**TM** **60 °C**	**TM** **70 °C**	**HI** **19.7**	**HI** **12.8**	**HI** **9.2**	**HI** **7.0**	**HI** **4.7**	**TM** **8.1**	**TM** **5.7**
C10:0	0.24	0.22	0.23	0.24	0.34	0.29	0.23	0.21	0.45	1.19	1.03	1.17	1.27	0.67	1.10
C12:0	46.7	46.8	50.2	49.1	0.33	0.29	0.26	0.26	36.3	35.9	43.9	32.8	34.4	0.39	0.33
C14:0	10.5	10.7	11.5	11.4	3.31	3.15	3.11	3.04	8.37	10.2	10.1	8.34	8.50	2.33	2.86
C16:0	12.9	13.6	14.8	14.5	18.6	17.8	17.8	17.6	16.6	19.2	16.5	19.2	17.3	19.4	16.8
C16:1 *c*9	2.17	2.17	2.17	2.14	1.53	1.48	1.47	1.46	3.07	2.69	3.57	3.01	4.67	0.92	1.40
C18:0	3.00	3.23	3.52	3.44	3.52	3.33	3.40	3.34	3.12	4.51	3.24	4.26	3.37	5.91	4.61
C18:1 *c*9	10.6	11.0	11.0	10.6	36.9	36.3	36.3	36.2	12.8	18.3	13.6	21.0	16.6	33.9	38.7
C18:2 n-6	10.9	9.5	4.42	6.07	31.4	33.3	33.3	33.7	16.4	5.82	5.48	6.96	10.4	31.8	30.6
C18:2 *c*9*t*11^c^	0.61	0.43	0.18	0.26	0.03	0.03	0.04	0.02	0.38	0.06	0.24	0.08	0.31	0.06	0.04
C18:3 n-3	0.78	0.64	0.23	0.38	1.41	1.59	1.55	1.64	1.18	0.28	0.47	0.45	1.12	1.40	1.32
Other^d^	1.60	1.71	1.75	1.87	2.63	2.44	2.54	2.53	1.33	1.85	1.87	2.73	2.06	3.22	2.24
Total SFA	74.0	75.2	80.8	79.2	26.6	25.3	25.4	25.0	65.6	71.7	75.6	66.7	65.9	29.6	26.3
Total BCFA	0.56	0.65	0.57	0.63	0.93	0.89	0.85	0.86	0.22	0.48	0.38	0.77	0.40	0.72	0.68
Total MUFA	13.8	14.3	14.0	13.7	39.7	38.9	38.9	38.8	16.6	21.8	18.1	25.0	22.2	36.5	41.1
Total PUFA	11.7	9.91	4.66	6.45	32.8	34.9	34.9	35.4	17.6	6.10	5.95	7.46	11.6	33.2	32.0
Total FA, g/kg DM	226	235	201	218	219	213	243	208	143	71.2	75.8	58.0	45.5	78.5	56.5

*FA* Fatty acids, *HI Hermetia illucens*, *TM Tenebrio molitor*, *c cis*, *SFA* Saturated fatty acids, *BCFA* Branched-chain fatty acids, *MUFA* Monounsaturated fatty acids, *PUFA* Polyunsaturated fatty acids, *DM* Dry matter

^a^Full-fat insect meals obtained applying different drying temperatures: HI meals processed at 40 °C (HI 40 °C), 50 °C (HI 50 °C), 60 °C (HI 60 °C) and 70 °C (HI 70 °C); TM meals processed at 30 °C (TM 30 °C), 50 °C (TM 50 °C), 60 °C (TM 60 °C) and 70 °C (TM 70 °C)

^b^Defatted insect meals containing different residual ether extract (EE) content: HI meals containing 19.7 (HI 19.7), 12.8 (HI 12.8), 9.2 (HI 9.2), 7.0 (HI 7.0) and 4.7 (HI 4.7) g EE/100g DM; TM meals containing 8.1 (TM 8.1) and 5.7 (TM 5.7) g EE/100g DM

^c^Coeluted with C18:2 *t*8*c*10 and C18:2 *t*7*c*9

^d^Other: C14:1 *c*9 + C15:0 + C15 *iso* + C15 *aiso* + C16 *iso* + C17:1 *c*9 + C17 *iso* + C17 *aiso* + ∑C18:1 *t* + C18:1 *c*11 + C18:1 *c*12 + C18:1 *c*14 + C18:1 *t*16 + C20:0 + C22:0 + C18:3 n-6 + C20:1 *c*9 + C20:1 *c*11 + C20:2 n-6 + C20:3 n-6 + C22:1 n-9 + C20:3 n-3 + C20:4 n-6

### Chemical composition of the rumen content

The CH_4_ production was quantified by gas chromatography (MicroGC Fusion, Inficon, East Syracuse, NY, USA), following the procedures described by Macheboeuf et al. [37]. Ammonia levels were determined using the Berthelot method [38]. The determination of VFA was performed according to Jouany [39] using a gas chromatograph (Perkin Elmer Clarus 580 GC, Perkin Elmer, Waltham, MA, USA) equipped with a capillary column (CP-WAX 58 FFAP 25 m × 0.25 mm, Agilent, Santa Clara, CA, USA). The organic matter (OM) content of the residue containing non-degraded particles was determined, and the in vitro organic matter disappearance (IVOMD) was calculated, as described by Renna et al. [25].

The procedures described by Alves et al. [40] were applied to determine the FA profile of the rumen content. The equipment and temperature program used, as well as peaks identification and quantification, were performed as detailed by Renna et al. [25].

### Calculations and statistical analyses

All data from the in vitro fermentation and rumen content were analysed using the SPSS software (version 27.0 for Windows; SPSS Inc., Chicago, IL, USA).

Data of full-fat HI and TM meals from Renna et al. [25] were integrated in the dataset of the defatted meals to test the effect of meal EE content on in vitro rumen digestibility and FA biohydrogenation. The full-fat HI and TM meals contained 26.9 and 39.2 g EE/100 g DM, respectively.

Regarding the in vitro fermentations, the total number of observations was 136 [17 meals × 4 incubation runs (statistical replications) × 2 technical replications (two bottles per sample and per incubation run)]. The results of the two technical repetitions were averaged for statistical analyses. By subtracting the results from blank cultures (ruminal buffered fluid without meal samples; two blanks per animal, with their average used for correction), the amount of feed degraded and the net production of fermentation end-products (VFA and ammonia) at the end of each incubation period were calculated.

A first GLM ANOVA was performed on the data of the full-fat meals obtained applying different drying temperatures, including insect species (HI and TM = 2 levels) as a fixed effect, animal (4 levels) as a random effect, temperature as a covariate and its interaction with insect species as a fixed effect.

A second GLM ANOVA was performed on the data of the defatted meals containing different residual EE content, including insect species (HI and TM = 2 levels) as a fixed effect, animal (4 levels) as a random effect, EE content as a covariate and its interaction with insect species as a fixed effect.

For both models, least squares means were reported with the pooled standard error of the mean (SEM) derived from the model. For each statistical model, data normality was verified using the Shapiro-Wilk test, while homogeneity of variances was visually verified with graphics of the residuals. Significance was declared at *P*-values < 0.05, while 0.05 ≤ *P*-values ≤ 0.10 were interpreted as a trend toward significance.

No statistical analysis was conducted to compare the chemical composition and FA profile of the tested meals as each meal derived from a single commercial batch.

## Results

### Chemical composition and fatty acid profile of the insect meals

#### Full-fat meals obtained applying different drying temperatures

The full-fat insect meals obtained applying different drying temperatures showed very similar chemical composition (Table 1) and FA profile (Table 2) within each insect species, while some variability was detected between the two species. In particular, the OM, CP and chitin contents were on average equal to 88.7, 35.6, 4.8 g/100 g DM for the full-fat HI meals, and 94.5, 45.1, 6.5 g/100 g DM for the full-fat TM meals.

The total FA content in the full-fat insect meals was always higher than 200 g/kg DM (Table 2). The proportion of total saturated fatty acids (SFA) in the full-fat HI meals was on average 77.3 g/100 g FA, while it was lower (on average 25.6 g/100 g FA) for the full-fat TM meals. In particular, the full-fat HI meals showed high proportions of medium-chain SFA, with the sum of lauric (C12:0), myristic (C14:0) and palmitic (C16:0) acids almost reaching three quarters of the total detected FA, and with lauric acid being by far the most abundant individual FA (about half of the total detected FA). On the contrary, the full-fat TM meals mainly contained oleic (C18:1 *c*9) and linoleic (C18:2 n-6) acids, followed by palmitic acid (on average 36.4, 32.9 and 18.0 g/100 g FA, respectively), resulting in higher total monounsaturated (MUFA) and total polyunsaturated fatty acids (PUFA) proportions when compared to the full-fat HI meals. All the tested full-fat insect meals contained very low proportions of rumenic (C18:2 *c*9*t*11; < 0.7 g/100 g FA) and α-linolenic (C18:3 n-3; < 1.7 g/100 g FA) acids, and did not contain long-chain n-3 PUFA.

### Defatted meals containing different residual ether extract contents

The chemical composition and FA profile of the defatted meals used in the trial are reported in Tables 1 and 2, respectively. The defatting process resulted in a CP content always higher than 41 and 56 g/100 g DM for the HI and TM meals, respectively. When compared to the full-fat meals of the same insect species used in the current trial and those published by Renna et al. [25] (HI: EE = 26.9 g/100 g DM, CP = 35.1 g/100 g DM; TM: EE = 39.2 g/100 g DM, CP = 33.9 g/100 g DM), the defatted meals showed lower EE (from 4.7 to 19.7 g/100 g DM for the defatted HI meals, while 5.7 and 8.1 g/100 g DM for the defatted TM meals) and higher CP contents (from 41.2 to 61.3 g/100 g DM for the defatted HI meals, while 56.5 and 63.8 g/100 g DM for the defatted TM meals). For both insect species, the CP content increased as far as the EE content decreased. The chitin content ranged from 5.5 to 8.4 g/100 g DM in the defatted HI meals and from 6.3 to 7.8 g/100 g DM in the defatted TM meals, also resulting numerically higher when compared to the values found for the full-fat meals reported by Renna et al. [25].

As far as the FA profile of the defatted insect meals is concerned, besides the expected lower total FA content (from 45 to 143 g/kg DM for the defatted HI meals and from 57 to 78 g/kg DM for the defatted TM meals) when compared to the full-fat meals used in this trial and those reported by Renna et al. [25] (209 and 317 g/kg DM for HI and TM, respectively), the proportions of the most abundant individual FA and groups of FA over the total detected FA remained substantially unchanged.

### Effect of drying temperature of full-fat insect meals

#### In vitro rumen fermentation parameters

The fermentation characteristics after 24 h of incubation of the full-fat insect meals obtained applying different drying temperatures are reported in Table 3. When expressed as mmol (or μmol)/g DM, the total gas and CH_4_ productions were not affected by insect species and drying temperature. Similar results were obtained when expressing CH_4_ as mmol/mmol of total gas.

**Table 3:** Effect of drying temperature of full-fat insect meals on in vitro rumen fermentation parameters

**Item**^**a**^	**Species**	**Intercept**	**Intercept SEM**	**Species coefficient**	**Species SEM**	**Temp coefficient**	**Temp SEM**	**Temp × Species coefficient**	**Temp × Species SEM**	***P*-value**			
										**Intercept**	**Species**	**Temp**	**Temp × Species**
Total gas 24 h, mmol/g DM		1.72	0.153	-	-	-	-	-	-	<0.001	0.377	0.205	0.065
CH_4_ 24 h, µmol/g DM		315	47.5	-	-	-	-	-	-	<0.001	0.643	0.149	0.584
CH_4_, mmol/mmol total gas		0.182	0.010	-	-	-	-	-	-	<0.001	0.670	0.123	0.614
pH final		6.97	0.04	-	-	-	-	-	-	<0.001	0.484	0.583	0.669
Total VFA, mmol/g DM		4.77	0.126	-	-	−0.009	0.002	-	-	<0.001	0.855	0.001	0.079
Acetate, g/100g VFA	HI	55.1	0.784	2.86	0.784	0.051	0.014	-	-	<0.001	0.001	0.001	0.075
	TM			−2.86									
Propionate, g/100g VFA	HI	16.8	0.459	−3.30	0.459	-	-	0.052	0.008	<0.001	<0.001	0.602	<0.001
	TM			3.30				−0.052					
Butyrate, g/100g VFA	HI	12.0	0.382	2.67	0.382	−0.028	0.069	-	-	<0.001	<0.001	<0.001	0.280
	TM			−2.67				-					
Acetate:Propionate ratio	HI	3.37	0.118	0.79	0.118	-	-	−0.112	0.002	<0.001	<0.001	0.721	<0.001
	TM			−0.79				0.112					
IVOMD, g/100g	HI	32.9	1.381	−7.44	1.381	-	-	0.054	0.025	<0.001	<0.001	0.895	0.042
	TM			7.44				−0.054					
NH_3_-N, mmol/g DM	HI	3.57	0.104	−0.50	0.104	−0.009	0.002	-	-	<0.001	<0.001	<0.001	0.904
	TM			0.50									
NH_3_-N, g/100 g total N		57.7	1.660	-	-	−0.126	0.030	-	-	<0.001	0.214	<0.001	0.914

*HI Hermetia illucens*, *TM Tenebrio molitor*, *SEM* Standard error of the mean, *Temp* Drying temperature expressed as °C, *DM* Dry matter, *VFA* Volatile fatty acids, *IVOMD* In vitro organic matter disappearance

^a^The missing coefficients in the table (-) correspond to non significant effects, and thus are considered equal to zero

The total VFA production decreased by 0.009 mmol/g DM for each additional 1 °C of drying temperature, irrespective of insect species. The acetate and butyrate proportions were significantly influenced by the drying temperature: the former increased (+0.051 g/100 g VFA) whereas the latter decreased (−0.028 g/100 g VFA) with 1 °C increase of the applied drying temperature. The effect of the interaction between drying temperature and species was significant for propionate proportion, which increased and decreased by increasing temperature for HI and TM meals, respectively.

The fermentation resulted in higher IVOMD with TM. Moreover, IVOMD slightly increased (+0.054 g/100 g for HI) or decreased (−0.054 g/100 g for TM) with 1 °C increase of the applied drying temperature.

The total ammonia production was negatively affected by the drying temperature (−0.009 mmol/g DM for each additional 1 °C, irrespective of insect species). When expressed as a proportion of total N, ammonia production was also affected by the drying temperature, being reduced by 0.126 g/100 g total N for each additional 1 °C.

### Fatty acid profile of rumen digesta

The main individual FA and groups of FA of rumen digesta after 24 h of incubation of full-fat HI and TM meals, which were significantly affected by the applied drying temperature or by the interaction between drying temperature and insect species, are reported in Table 4. The other individual FA and groups of FA are instead reported in Additional file 1.

**Table 4:** Effect of drying temperature of full-fat insect meals on the fatty acid profile of rumen digesta, g/100 g total FA^a^

**Item**^**b**^	**Species**	**Intercept**	**Intercept SEM**	**Species coefficient**	**Species SEM**	**Temp coefficient**	**Temp SEM**	**Temp ×** **Species** **coefficient**	**Temp ×** **Species** **SEM**	***P*-value**			
										**Intercept**	**Species**	**Temp**	**Temp ×** **Species**
C14:0	HI	8.38	0.306	2.18	0.306	-	-	0.02	0.006	<0.001	<0.001	0.639	0.001
	TM			−2.18				−0.02					
C16:1 *c*9	HI	1.35	0.106	0.30	0.106	0.004	0.002	-	-	<0.001	0.009	0.034	0.953
	TM			−0.30									
C18:2 n-6	HI	8.43	1.761	-	-	-	-	−0.07	0.031	<0.001	0.421	0.845	0.043
	TM							0.07					
Total SFA	HI	64.16	1.788	14.34	1.788	-	-	0.07	0.032	<0.001	<0.001	0.489	0.043
	TM			−14.34				−0.07					
Total PUFA	HI	10.47	1.793	-	-	-	-	−0.07	0.032	<0.001	0.275	0.751	0.040
	TM							0.07					
Total n-6 FA	HI	0.873	0.061	−0.322	0.061	-0.004	0.001	-	-	<0.001	<0.001	0.002	0.100
	TM			0.322									

*FA* Fatty acids, *HI Hermetia illucens*, *TM Tenebrio molitor*, *SEM* Standard error of the mean, *Temp* Drying temperature expressed as °C, *c cis*, *SFA* Saturated fatty acids, *PUFA* Polyunsaturated fatty acids

^a^Only main individual FA and groups of FA showing a significant temperature or temperaure × species interaction effect in the GLM ANOVA are reported in Table 4. The detailed FA profile is reported in Additional file 1

^b^The missing coefficients in the table (-) correspond to non significant effects, and thus are considered equal to zero

Only few FA were affected, marginally, by the drying temperature. The proportion of C16:1 *c*9 increased by 0.004 g/100 g FA whereas that of total n-6 FA decreased by 0.004 g/100 g FA for each additional 1 °C, irrespective of insect species. The proportion of total SFA in rumen digesta significantly increased by 0.07 g/100 g FA for each additional 1 °C when the HI meals were incubated, whereas it decreased by the same amount with the TM meals. The concentration of C14:0 increased by 0.02 g/100 g FA for each additional 1°C and decreased by the same amount when HI and TM meals were incubated, respectively. A decreasing and increasing rate of −0.07 and +0.07 g/100 g FA for each additional 1 °C of drying temperature were observed for C18:2 n-6 and total PUFA proportions in rumen digesta when HI and TM meals were incubated, respectively.

### Effect of the ether extract content of insect meals

#### In vitro rumen fermentation parameters

The fermentation characteristics obtained after 24 h of incubation of the insect meals containing different EE contents are shown in Table 5.

**Table 5:** Effect of ether extract content of insect meals on in vitro rumen fermentation parameters

**Item**^**a**^	**Species**	**Intercept**	**Intercept** **SEM**	**Species** **coefficient**	**Species** **SEM**	**EE** **coefficient**	**EE** **SEM**	**EE ×** **Species** **coefficient**	**EE ×** **Species** **SEM**	***P*-value**			
										**Intercept**	**Species**	**EE**	**EE ×** **Species**
Total gas 24 h, mmol/g DM		3.02	0.089	-	-	−0.038	0.005	-	-	<0.001	0.912	<0.001	0.266
CH_4_ 24 h, µmol/g DM		743	26.700	-	-	−12.9	1.460	-	-	<0.001	0.597	<0.001	0.133
CH_4_, mmol/mmol total gas	HI	0.253	0.0041	-	-	−0.002	0.0002	−0.0006	0.0002	<0.001	0.140	<0.001	0.017
	TM							0.0006					
pH final		6.95	0.02	-	-	-	-	-	-	<0.001	0.524	0.495	0.705
Total VFA, mmol/g DM		5.83	0.105	-	-	−0.063	0.006	-	-	<0.001	0.601	<0.001	0.116
Acetate, g/100g VFA	HI	62.3	0.300	1.66	0.016	−0.18	0.016	−0.045	0.016	<0.001	<0.001	<0.001	0.009
	TM			−1.66				0.045					
Propionate, g/100g VFA		15.4	0.220	-	-	0.122	0.012	-	-	<0.001	0.983	<0.001	0.517
Butyrate, g/100g VFA	HI	7.58	0.200	-	-	0.088	0.011	0.055	0.011	<0.001	0.367	<0.001	<0.001
	TM							−0.055					
Acetate:Propionate ratio		4.0	0.057	-	-	−0.033	0.003	-	-	<0.001	0.092	<0.001	0.492
IVOMD, g/100g	HI	45.9	0.966	2.41	0.966	−0.57	0.053	−0.209	0.053	<0.001	0.018	<0.001	<0.001
	TM			−2.41				0.209					
NH_3_-N, mmol/g DM	HI	3.94	0.072	−0.23	0.072	−0.036	0.004	-	-	<0.001	0.004	<0.001	0.962
	TM			0.23									
NH_3_-N, g/100 g total N		49.7	1.310	-	-	-	-	-	-	0.001	0.132	0.456	0.190

*HI Hermetia illucens*, *TM Tenebrio molitor*, *SEM* Standard error of the mean, *EE* Ether extract expressed as g/100 g DM, *DM* Dry matter, *VFA* Volatile fatty acids, *IVOMD* In vitro organic matter disappearance

^a^The missing coefficients in the table (-) correspond to non significant effects, and thus are considered equal to zero

The total gas and CH_4_ productions did not vary between the insect species, but they were reduced by 0.038 mmol/g DM for total gas and 12.9 µmol/g DM for CH_4_ for each additional 1 g EE/100 g DM content of the meals. Similarly, the total VFA production decreased by 0.063 mmol/g DM for each additional 1 g EE/100 g DM content of the meals, irrespective of insect species. When expressed as mmol/mmol of total gas, CH_4_ also decreased when the EE content of the insect meals increased, this reduction being more pronounced for HI than TM meals.

The acetate proportion was affected by insect species, values for TM being lower than values for HI. The proportion of the different VFA varied with the EE content of the meals: propionate and butyrate increased whereas acetate decreased while increasing the EE content. The reduction of the acetate proportion and the increase of the butyrate proportion were both more pronounced when HI than TM meals were incubated.

The IVOMD decreased while increasing the EE content of the meals, this reduction being more pronounced when the HI rather than the TM meals were incubated (−0.78 and −0.36 g/100 g for each additional 1 g EE/100 g DM content of the meals, respectively).

The total ammonia production was higher when the TM meals rather than the HI meals were incubated, and decreased by 0.036 mmol/g DM for each additional 1 g EE/100 g DM content of the meals. When expressed as a proportion of total N, the ammonia production did not vary with insect species or EE content of the meals.

### Fatty acid profile of rumen digesta

The main individual FA and groups of FA of rumen digesta that, after 24 h of incubation of the HI and TM meals, were significantly affected by insect species, EE content of the meals and/or their interaction, are reported in Table 6. The other individual FA and groups of FA are instead reported in Additional file 2.

**Table 6:** Effect of ether extract content of insect meals on the fatty acid profile of rumen digesta, g/100 g total FA (unless otherwise stated)^a^

**Item**^**b**^	**Species**	**Intercept**	**Intercept** **SEM**	**Species** **coefficient**	**Species** **SEM**	**EE** **coefficient**	**EE** **SEM**	**EE ×** **Species** **coefficient**	**EE x** **Species** **SEM**	***P*-value**			
										**Intercept**	**Species**	**EE**	**EE ×** **Species**
C12:0	HI	11.72	1.370	10.88	1.370	-	-	-	-	<0.001	<0.001	0.574	0.517
	TM			−10.88									
C14:0	HI	6.77	0.274	2.14	0.274	-	-	-	-	<0.001	<0.001	0.081	0.440
	TM			−2.14									
C15:0		1.86	0.105	-	-	−0.029	0.011	-	-	<0.001	0.770	<0.001	0.399
C18:0	HI	10.76	0.566	−2.53	0.566	−0.09	0.031	-	-	<0.001	<0.001	0.005	0.176
	TM			2.53									
C13 iso	HI	0.335	0.021	−0.086	0.021	−0.006	0.0012	-	-	<0.001	<0.001	<0.001	0.130
	TM			0.086									
C14 iso	HI	1.544	0.064	-	-	−0.028	0.004	-	-	<0.001	0.188	<0.001	0.577
	TM												
C15 iso		2.47	0.128	-	-	−0.04	0.007	-	-	<0.001	0.260	<0.001	0.455
C15 aiso		3.87	0.193	-	-	−0.07	0.011	-	-	<0.001	0.290	<0.001	0.445
C16 iso		1.33	0.057	-	-	−0.02	0.003	-	-	<0.001	0.402	<0.001	0.890
C17 iso		0.453	0.030	-	-	−0.007	0.0017	-	-	<0.001	0.849	<0.001	0.983
C17 aiso		1.30	0.070	-	-	−0.02	0.004	-	-	<0.001	0.246	<0.001	0.813
C16:1 *c*9	HI	1.38	0.131	0.86	0.131	-	-	−0.026	0.007	<0.001	<0.001	0.112	0.001
	TM			−0.86				0.026					
C18:1 *c*9 (+*c*10+*t*15)	HI	13.27	0.738	−1.56	0.738	0.09	0.040	−0.23	0.040	<0.001	0.043	0.039	<0.001
	TM			1.56				0.23					
C18:2 n-6	HI	10.78	0.764	−7.31	0.764	0.12	0.042	-	-	<0.001	<0.001	0.007	0.062
	TM			7.31									
CLA *c*9*t*11 (+*t*7*c*9+*t*8*c*10)	HI	-	-	-	-	0.011	0.001	−0.005	0.001	0.079	0.175	<0.001	0.020
	TM							0.005					
C18:3 n-3	HI	1.25	0.066	−0.167	0.066	−0.01	0.004	-	-	<0.001	0.017	0.022	0.257
	TM			0.167									
Total SFA	HI	58.52	1.392	10.01	1.392	−0.18	0.076	-	-	<0.001	<0.001	0.027	0.107
	TM			−10.01									
Total OCFA		2.72	0.186	-	-	−0.04	0.010	-	-	<0.001	0.077	<0.001	0.383
Total BCFA		11.46	0.514	-	-	−0.19	0.028	-	-	<0.001	0.081	<0.001	0.495
Total MUFA	HI	17.73	0.784	-	-	0.14	0.043	−0.23	0.043	<0.001	0.071	0.002	<0.001
	TM							0.23					
Total PUFA	HI	13.11	0.794	−7.42	0.794	0.12	0.043	-	-	<0.001	<0.001	0.013	0.082
	TM			7.42									
Total n-3 FA		1.58	0.069	-	-	−0.02	0.004	-	-	<0.001	0.206	<0.001	0.948
Total n-6 FA	HI	0.895	0.046	−0.118	0.046	−0.005	0.003	-	-	<0.001	0.015	0.048	0.197
	TM			0.118									
Total FA, g/kg DM		2006	234	-	-	50.62	12.78	-	-	<0.001	0.411	<0.001	0.277

*FA* Fatty acids, *HI Hermetia illucens*, *TM Tenebrio molitor*, *SEM* Standard error of the mean, *EE* Ether extract expressed as g/100g DM, *c cis*, *t trans*, *SFA* Saturated fatty acids, *OCFA* Odd-chain fatty acids, *BCFA* Branched-chain fatty acids, *MUFA* Monounsaturated fatty acids, *PUFA* Polyunsaturated fatty acids, *DM* Dry matter

^a^Only main individual FA and FA groups showing a significant species, EE or species × EE effect in the GLM ANOVA are reported in Table 6. The detailed FA profile is reported in Additional file 2

^b^The missing coefficients in the table (-) correspond to non significant effects, and thus are considered equal to zero

The proportion of total SFA in rumen digesta was higher when HI rather than TM meals were incubated, and decreased by 0.18 g/100 g FA for each additional 1 g EE/100 g DM, irrespective of insect species. The C12:0 and C14:0 proportions were significantly higher in rumen digesta when HI rather than TM meals were incubated, but they were not affected by the EE content of the meals. The C18:0 proportion in the rumen digesta was lower when HI rather than TM meals were incubated, and showed a significant decrease by 0.09 g/100 g FA for each additional 1 g EE/100 g DM, irrespective of insect species. The total odd-chain fatty acids (OCFA) and the total BCFA proportions in rumen digesta were unaffected by the insect species; whatever the insect species, their proportions decreased by 0.04 g/100 g FA and 0.19 g/100 g FA for each additional 1 g EE/100 g DM, respectively. The same decreasing trend was observed for the detected individual odd- and branched-chain FA.

The proportion of total MUFA in rumen digesta significantly decreased by 0.09 g/100 g FA and increased by 0.38 g/100 g FA for each additional 1 g EE/100 g DM when HI and TM meals were incubated, respectively. A similar trend was observed for C16:1 *c*9. The proportion of 18:1 *c*9 (+*c*10+*t*15) in rumen digesta increased with the increase of the EE content with both insect species, but such increase was slower with HI than with TM (+0.14 vs. +0.32 g/100 g FA for each additional 1 g EE/100 g DM, respectively).

The incubation of HI meals resulted in lower proportions of total PUFA in the rumen digesta when compared to the incubation of TM meals. Moreover, the proportions of both total PUFA and C18:2 n-6 increased by 0.12 g/100 g FA and the proportion of C18:3 n-3 decreased by 0.01 g/100 g FA for each additional 1 g EE/100 g DM, irrespective of insect species.

## Discussion

### Effect of drying temperature of full-fat insect meals

#### Chemical composition and in vitro rumen fermentation parameters

Post killing stages of insect meals production may encompass different drying techniques, such as hot-air drying, freeze-drying, sun-drying, and microwave-drying [41]. Hot-air drying is the most used drying method by the insect industry because it is the least expensive and can be easily adapted to continuous industrial operations [41, 42], allowing the preservation of color, protein and fat quality [43]. Commonly applied oven-drying temperatures range from 50 to 80 °C [10, 16]. Few data are currently available on the effects of processing on the nutritional composition of edible insects; moreover, published information has focused more on comparing processing methods (e.g., [42, 44]) rather than comparing distinct sets of temperatures for each drying methodology. When focusing on hot-air drying, it has been shown that processing black crickets (*Gryllus bimaculatus* De Geer) at 120 °C rather than at 45 °C determined a lowering by 1% of total weight in their protein content [45], which seems to be also in line with the results obtained by Aniebo and Owen [46] for housefly (*Musca domestica* L.) larvae. On the contrary, the CP content of the full-fat HI and TM meals used in our study was very similar after applying different drying temperatures, such result most probably being imputable to less variability and lower maximum temperature (from 30 °C to 70 °C) reached in our study.

In our study, the tested drying temperatures had limited effects on in vitro ruminal fermentation parameters, which is in agreement with the similarity of the chemical composition of the full-fat insect meals. Total gas and CH_4_ productions after 24 h of incubation were not significantly affected, whereas the total VFA production slightly decreased while increasing the drying temperature. Such reduction was unexpected as total gas and total VFA productions are generally closely correlated [47], but it has to be pointed out that the slope coefficient was very low. The IVOMD also remained unaltered by the drying temperature. The low drying temperatures we used, would have probably prevented important physicochemical modifications of fermentable constituents [48].

Ammonia production significantly decreased while increasing the applied drying temperature, both when expressed as mmol/g DM and as a proportion of total N. Although variations in ammonia were low, the obtained results imply that drying temperature may have led to a reduction of in vitro degradation of proteins from insect meals. In fact, ammonia production obtained after in vitro rumen fermentation constitutes an indicator of ruminal protein degradation [49]. On one hand, heating temperature is known to reduce protein solubility and digestibility due to protein oxidation, Maillard reactions or protein denaturation [50]. Azzollini et al. [43] reported that insect proteins can undergo structural changes, such as denaturation or interaction with lipids, during blanching and drying processes. Kröncke et al. [51] observed a reduction in protein solubility when TM larvae were dried at 60 °C and 120 °C, the protein solubility being the lowest at 120 °C. On the other hand, heating temperature can limit ruminal protein degradation [52], increasing the intestinal protein flow, which can be favorable for ruminant production provided that intestinal digestibility is high. Thus, as previously mentioned in Renna et al. [25], insect meals would be sources of rumen undegradable protein. However, heat treatment may sometimes reduce intestinal protein digestibility [50], this effect being temperature-dependent, as observed by Huang et al. [53]: hot air drying (at 60 °C) of HI meal showed better protein digestibility than microwave drying, the lower temperature of conventional oven-drying limiting protein damage.

### Fatty acid profile of insect meals and rumen digesta

Dobermann et al. [45] reported major effects of drying temperature on the FA profile of black crickets, as processing at 120 °C rather than at 32 °C, 45 °C or 72 °C determined a reduction of C18:2 n-6 and C18:3 n-3 contents, a result that was attributed by these authors to PUFA sensitivity to oxidation with temperatures above 60 °C. Even if no statistical analysis was conducted to our full-fat meals processed at different temperatures, overall the obtained chemical composition and FA profile showed very similar values within species, suggesting that the applied temperatures were too low to determine a noteworth change in their nutritional composition, which is in line with the findings obtained by Dobermann et al. [45]. Also, when considering the effects of heating on the FA profile of other food and feed products, published literature clearly shows that significant changes are usually seen at heating temperatures higher than those used in our study; while heating, lipids can undergo chemical reactions including oxidation, hydrolysis, polymerization, isomerization, and cyclization, which impair FA stability [54]. On the contrary, and consistently with our findings, heating milk during the cheesemaking process, within a similar temperature range used in our study, was found not to affect the milk FA profile [55, 56].

In rumen digesta, a reduction in the proportion of PUFA in favour to a small increase of SFA while increasing the drying temperature could have been hypotesized, especially considering the lower fat melting point and the higher susceptibility of PUFA to oxidation [57]. However, we detected only negligible effects of the drying temperature on the FA profile of rumen digesta, which has to be attributed to the low applied temperatures, that had no effects on the chemical composition and FA profile of the meals. Even if the proportions of some few minor individual FA or groups of FA in the rumen digesta were affected by the drying temperature applied to obtain the insect meals (Table 4 and Additional file 1), the slope coefficients were in all the cases very low. Their variation due to the drying temperature between the minimum and maximum value of the studied temperature range was almost equivalent to the SEM of the model and had negligible effects on the intercept of the model itself.

## Effect of ether extract content of insect meals

### Chemical composition and in vitro rumen fermentation parameters

The chemical composition of the defatted HI and TM meals tested in the current study fell within the wide ranges currently reported in published reviews [58, 59]. Defatted meals are characterized by much higher compositional variability when compared to full-fat meals from the same insect species, as clearly observed analysing the range values recently reported by Gasco et al. [4]. Such observation derives from the lack of standardized defatting technologies which, as already mentioned, resolve in poor consistency of the chemical composition of the meals.

Overall, defatting increases the concentration of the other main constituents, including the protein and chitin contents [4, 60]. Lu et al. [59] reported an average CP content of HI larvae equal to 414.7 g/kg DM, which was lower than that of a conventional soybean meal (494.4 g/kg DM). The same authors reported that, after defatting, values of CP in HI larvae up to 655 g/kg DM can be obtained, such value being only slightly higher than that found in our HI 4.7 meal (613 g/kg DM).

Regarding the EE content, in full-fat meals very high values can be obtained (e.g., up to 515.3 g/kg DM for HI larvae [59]). Even after defatting, the EE content of insect meals reach values that are higher than those typically found in plant meals commonly used in ruminant nutrition (e.g., 69.2 vs. less than 20 g/kg DM for HI larvae and soybean meal, respectively; [59, 61]), which can have an impact on feed digestibility and animal performance [24, 62].

Defatting of insect meals increased the ruminal fermentation process, as shown by the significant increase in total gas and VFA productions. High fat content is known to inhibit rumen microbes and decrease the carbohydrates digestibility [62]. Compared to full-fat HI and TM meals [25], both the total gas and VFA productions were increased. Consequently, also the IVOMD increased with the reduction of the EE content of the meals. This improvement in DM and OM degradability and total gas production, after either mechanical or chemical defatting, was also observed in vitro by Mulianda et al. [63] when replacing full-fat with defatted HI meals. As already hypothesized by Renna et al. [24], the obtained results show that defatted insect meals are more suitable to be used in ruminant nutrition when compared to full-fat ones. It should also be noted that the improvement in VFA production, DM and OM degradability was expected due to the non-degradability of lipids in the rumen [64], which can penalize in vitro evaluations of full-fat meals, even though they could be valuable dietary ingredients. In fact, lipids are not degraded by rumen microbes as readily as carbohydrates. High lipid levels can coat feed particles, limiting microbial access to digestible carbohydrates and other nutrients [65]. This coating effect can lead to an underestimation of DM and OM degradability in in vitro evaluations.

Defatting of insect meals also increased the total CH_4_ production, first as a result of the increased fermentation process. Moreover, the reduction in the EE content, and also in specific FA (i.e., C12:0, C14:0, or UFA) which are known to reduce CH_4_ production [66, 67], may have contributed to such increase.

Defatting of insect meals increased the ammonia production as well. This was the direct consequence of the increase of the CP content in insect meals following the fat removal. However, when expressing ammonia as a proportion of total incubated N, no differences were observed between the full-fat and defatted insect meals, confirming that the apparent rate of protein degradation is similar among them [25], even if an uptake also by rumen bacteria cannot be excluded.

### Fatty acid profile of insect meals and rumen digesta

As seen for the chemical composition, also the FA profile of the defatted HI and TM meals resembles available published data [58, 59].

The observed outcomes of insect meals defatting on the FA profile of rumen digesta were expected, and mainly imputable to the mitigation of the well known inhibitory effects that fat exerts on the ruminal microflora [68]. The first of such expected outcomes was the observed significant decrease of the proportion of total PUFA, and the contemporarily significant increase of C18:0 (irrespective of insect species), in rumen digesta while decreasing the EE content of the meals. This is the consequence of a higher PUFA disappearance (biohydrogenation rate) inside the rumen, which also leads to an increase of C18:0 as end product of this metabolic process [69]. By lowering the EE content of the insect meals, defatting lessens the inhibitory action exerted by fat on the ruminal microflora responsible for the biohydrogenation of unsaturated FA, which is well established in published literature [70].

The second clear effect of insect meals defatting was the increase of the proportions of odd- and branched-chain FA (i.e., C15:0, C14 iso, C15 iso, C15 aiso, C16 iso, C17 aiso, total OCFA, and total BCFA) in rumen digesta. These FA are of microbial origin, being prevalently synthesized by rumen bacteria starting from amino acids (i.e., leucine, isoleucine and valine) [71]. They have been successfully used as noninvasive biomarkers of rumen function under different dietary conditions, and are known to possess nutritional benefits for humans [72]. Lowering the inhibitory effects of fat, and especially of unsaturated C18 FA levels, leads to an increase of rumen bacteria and, consequently, to an increase of the proportion FA of microbial origin in rumen digesta.

In addition, while decreasing the EE content of the meals, the C18:1 *c*9 and the total MUFA proportions increased and decreased while incubating HI and TM meals, respectively, which seems to be the direct consequence of the noticeable differences in the FA profile of the two considered insect species (Table 2), as previously outlined.

### Effect of insect species

The in vitro ruminal incubation of the full-fat HI and TM meals of our study resulted in small differences between insect species for few fermentation parameters, only. Similar results were obtained when incubating our defatted HI and TM meals, and confirm previous findings [25].

The lack of a significant effect of insect species on CH_4_ production was expected. In fact, both medium-chain SFA (mainly C12:0 and C14:0) and unsaturated FA (UFA) have been shown to possess antimethanogenic effects [66, 67]. In this sense, the much higher proportion of the sum C12:0 + C14:0 found in full-fat HI meals (on average, 59.2 g/100 g FA) when compared to full-fat TM meals (on average, 3.44 g/100 g FA) was most probably counterbalanced by the much higher UFA proportion found in full-fat TM meals (on average, 73.6 g/100 g FA) when compared to full-fat HI meals (on average, 21.1 g/100 g FA). The same counterbalancing can be hypotesised for defatted meals, having the defatted HI meals higher proportion of the sum C12:0 + C14:0 (on average, 45.8 g/100g FA) compared to the defatted TM meals (on average, 2.96 g/100g FA), but lower UFA proportion (71.4 vs. 30.5 g/100 g FA, respectively). As a confirmation, published literature has shown antimethanogenic effects for both HI and TM oils, mainly as a result of the reduction in nutrient digestibility with both lipid sources [73]. Another factor limiting CH_4_ production could be the presence of chitin in insect meals [74]. The quite similar chitin content of the meals obtained from the two insect species considered in our study are in agreement with the the lack of a significant effect of insect species on CH_4_ production.

Insect species showed a significant effect on ammonia production, when the latter was expressed as mmol/g DM. Both statistical models showed higher ammonia production when TM rather than HI meals were incubated (Tables 3 and 5), which may be related to the higher CP content found in TM (on average, 45.1 and 60.2 g/100 g DM for the full-fat and defatted meals) when compared to HI (on average, 35.6 and 51.1 g/100 g DM); in fact, part of CP is degraded inside the rumen to amino acids, and subsequently to ammonia, by the proteolytic microflora [75].

As far as the FA profile of rumen digesta is concerned, major effects of insect species were observed for C12:0, C14:0, C18:0, C18:1 *c*9, C18:2 n-6, total SFA, total PUFA and total n-6 PUFA proportions. Results were quite consistent with both statistical models (Tables 4 and 6), this being the consequence of the lack of significant differences in the FA profiles of full-fat and defatted meals obtained from the same species, as found in our study and also recently pointed out by Toral et al. [76]. The higher proportions of C12:0 and total SFA when HI rather than TM meals were incubated were expected outcomes. Considering that SFA entering the rumen are not further metabolized in this organ [77], the obtained results are the direct consequence of the noticeable C12:0 content naturally present in HI larvae [78]. The incubation of TM meals instead resulted in higher C18:0 proportions in the rumen digesta when compared to HI meals. Such result is imputable to the higher UFA proportions typical of TM rather than HI meals [76]; in fact, most UFA entering the rumen undergo a biohydrogenation process which has, as final end product, C18:0 [69]. Finally, the higher proportions of C18:1 *c*9, C18:2 n-6, total PUFA and total n-6 FA found in the rumen digesta when TM rather than HI meals were incubated are the direct consequence of the higher proportions found for these FA in TM when compared to HI meals. A part of the unsaturated FA entering the rumen normally escapes ruminal biohydrogenation, especially with high fat content [79], thus explaining our findings.

The above-mentioned modifications of the FA of rumen digesta related to the effect of insect species should be taken into consideration carefully when ruminant nutrition is aimed at improving the concentration of health-promoting FA in derived food products, such as milk and meat [24, 25, 76].

## Conclusions

A drying temperature of full-fat HI and TM meals in the range from 30 to 70 °C had only minor effects on in vitro ruminal digestibility and FA profile of rumen digesta. However, increasing the drying temperature led to a slight reduction of in vitro degradation of proteins from insect meals, as outlined by the significant decrease in the ammonia production. Potentially, this may imply an increase of the intestinal protein flow, which could be favorable for ruminant production. Further protein evaluations will be required to assess the intestinal digestibility of proteins from insect meals to determine their true potential as rumen undegradable protein sources.

By reducing the inhibitory effects that fat exert on the rumen microbiota, defatting increased the in vitro ruminal digestibility of both HI and TM meals, as shown by the significant increase in total gas and VFA productions. However, as a counterbalance, also CH_4_ production increased, both as a result of the increased fermentation process and the reduction of total fat and specific FA that are known to reduce methanogenesis. Also, defatting decreased the proportion of PUFA and increased those of SFA and BCFA in ruminal digesta, which can have implications of the quality of the lipid fraction of milk and meat.

## Supplementary Information

Additional file 1: Table S1. Effect of drying temperature of full-fat insect meals on detailed fatty acid profile of rumen digesta, g/100 g total FA.

Additional file 2: Table S2. Effect of ether extract content of insect meals on detailed fatty acid profile of rumen digesta, g/100 g total FA.

## Data Availability

The datasets analysed in the current study are available from the corresponding author on request.

## Abbreviations

ADF: Acid detergent fibre
ADIN: Acid detergent insoluble nitrogen
ADL: Acid detergent lignin
BCFA: Branched-chain fatty acids
*c*: cis
CP: Crude protein
DM: Dry matter
EE: Ether extract
FA: Fatty acids
HI: *Hermetia illucens*
IVOMD: In vitro organic matter disappearance
MUFA: Monounsaturated fatty acids
NDF: Neutral detergent fibre
NDIN: Neutral detergent insoluble nitrogen
OCFA: Odd-chain fatty acids
OM: Organic matter
PUFA: Polyunsaturated fatty acids
SEM: Standard error of the mean
SFA: Saturated fatty acids
*t*: trans
TM: *Tenebrio molitor*
UFA: Unsaturated fatty acids
VFA: Volatile fatty acids
