# Supplementary material for: Low drying temperature has negligible impact but defatting increases in vitro rumen digestibility of insect meals, with minor changes on fatty acid biohydrogenation
Source: J Anim Sci Biotechnol. 2025 May 7;16:64. doi: 10.1186/s40104-025-01199-5 (PMC12056994; doi:10.1186/s40104-025-01199-5)
Supplement: Supplementary file 1 — Additional file 1: Table S1. Effect of drying temperature of full-fat insect meals on detailed fatty acid profile of rumen digesta, g/100 g total FA. [file 40104_2025_1199_MOESM1_ESM.docx]

## Table S1 Effect of drying temperature of full-fat insect meals on detailed fatty acid profile of rumen digesta, g/100 g total FA

| **Item^1^** | **Species** | **Intercept** | **Intercept**  **SEM** | **Species**  **coefficient** | **Species**  **SEM** | **Temp**  **coefficient** | **Temp**  **SEM** | **Temp ×**  **Species**  **coefficient** | **Temp ×**  **Species**  **SEM** | ***P*-value** | | | |
| --- | --- | --- | --- | --- | --- | --- | --- | --- | --- | --- | --- | --- | --- |
|  |  |  |  |  |  |  |  |  |  | **Intercept** | **Species** | **Temp** | **Temp ×**  **Species** |
| C10:0 | HI | 0.55 | 0.123 | 0.46  -0.46 | 0.123 | - | - | - | - | <0.001 | 0.001 | 0.600 | 0.650 |
|  | TM |  |  |  |  |  |  |  |  |  |  |  |  |
| C11:0 (+C10:1 *c*9) |  | 0.074 | 0.011 | - | - | -0.001 | 0.0002 | - | - | <0.001 | 0.583 | 0.017 | 0.895 |
|  |  |  |  |  |  |  |  |  |  |  |  |  |  |
| C12:0 | HI | 25.06 | 1.639 | 23.96 | 1.639 | - | - | - | - | <0.001 | <0.001 | 0.709 | 0.560 |
|  | TM |  |  | -23.96 |  |  |  |  |  |  |  |  |  |
| C13:0 |  | 0.0223 | 0.004 | - | - | -0.0002 | 0.00007 | - | - | <0.001 | 0.572 | 0.010 | 0.442 |
|  |  |  |  |  |  |  |  |  |  |  |  |  |  |
| C15:0 | HI | 0.93 | 0.113 | -0.39 | 0.113 | - | - | - | - | <0.001 | 0.020 | 0.299 | 0.431 |
|  | TM |  |  | 0.39 |  |  |  |  |  |  |  |  |  |
| C16:0 | HI | 19.46 | 0.992 | -6.72 | 0.992 | - | - | - | - | <0.001 | <0.001 | 0.217 | 0.307 |
|  | TM |  |  | 6.72 |  |  |  |  |  |  |  |  |  |
| C17:0 | HI | 0.269 | 0.022 | -0.093 | 0.022 | 0.002 | 0.0040 | - | - | <0.001 | <0.001 | 0.001 | 0.222 |
|  | TM |  |  | 0.093 |  |  |  |  |  |  |  |  |  |
| C18:0 | HI | 9.24 | 1.606 | -5.00  5.00 | 1.606 | - | - | - | - | <0.001 | 0.005 | 0.696 | 0.677 |
|  | TM |  |  |  |  |  |  |  |  |  |  |  |  |
| C20:0 | HI | 0.11 | 0.011 | -0.04  0.04 | 0.011 | - | - | - | - | <0.001 | 0.001 | 0.504 | 0.103 |
|  | TM |  |  |  |  |  |  |  |  |  |  |  |  |
| C21:0 (+CLA *t*9*c*11) |  | 0.022 | 0.005 | - | - | - | - | - | - | <0.001 | 0.209 | 0.887 | 0.217 |
|  |  |  |  |  |  |  |  |  |  |  |  |  |  |
| C22:0 |  | 0.034 | 0.006 | - | - | - | - | - | - | <0.001 | 0.177 | 0.302 | 0.974 |
|  |  |  |  |  |  |  |  |  |  |  |  |  |  |
| C13 iso | HI | 0.200 | 0.032 | -0.104 | 0.032 | -0.001 | 0.0057 | - | - | <0.001 | 0.003 | 0.020 | 0.086 |
|  | TM |  |  | 0.104 |  |  |  |  |  |  |  |  |  |
| C13 aiso |  | 0.04 | 0.0141 | - | - | - | - | - | - | 0.010 | 0.458 | 0.372 | 0.295 |
|  |  |  |  |  |  |  |  |  |  |  |  |  |  |
| C14 iso | HI | 0.74 | 0.129 | 0.31 | 0.129 | - | - | - | - | <0.001 | 0.023 | 0.186 | 0.311 |
|  | TM |  |  | -0.31 |  |  |  |  |  |  |  |  |  |
| C15 iso |  | 1.11 | 0.289 | - | - | - | - | - | - | 0.001 | 0.310 | 0.532 | 0.752 |
|  |  |  |  |  |  |  |  |  |  |  |  |  |  |
| C15 aiso |  | 1.91 | 0.451 | - | - | - | - | - | - | <0.001 | 0.079 | 0.336 | 0.513 |
|  |  |  |  |  |  |  |  |  |  |  |  |  |  |
| C16 iso |  | 0.57 | 0.152 | - | - | - | - | - | - | 0.001 | 0.889 | 0.889 | 0.360 |
|  |  |  |  |  |  |  |  |  |  |  |  |  |  |
| C17 iso | HI | 0.235 | 0.023 | -0.090 | 0.023 | - | - | 0.001 | 0.0004 | <0.001 | 0.001 | 0.477 | 0.016 |
|  | TM |  |  | 0.090 |  |  |  | -0.001 |  |  |  |  |  |
| C17 aiso |  | 0.56 | 0.140 | - | - | - | - | - | - | <0.001 | 0.106 | 0.818 | 0.549 |
|  |  |  |  |  |  |  |  |  |  |  |  |  |  |
| C18 iso | HI | 0.23 | 0.040 | -0.10 | 0.040 | - | - | - | - | <0.001 | 0.014 | 0.362 | 0.785 |
|  | TM |  |  | 0.10 |  |  |  | - |  |  |  |  |  |
| C12:1 *c*9 | HI | 0.155 | 0.015 | -0.088 | 0.015 | -0.001 | 0.0028 | - | - | <0.001 | <0.001 | 0.047 | 0.139 |
|  | TM |  |  | 0.088 |  |  |  |  |  |  |  |  |  |
| C14:1 *t*9 |  | 0.14 | 0.031 | - | - | - | - | - | - | <0.001 | 0.140 | 0.273 | 0.870 |
|  |  |  |  |  |  |  |  |  |  |  |  |  |  |
| C16:1 *c*7 | HI  TM | 0.72 | 0.159 | -0.42  0.42 | 0.159 | - | - | - | - | <0.001 | 0.014 | 0.498 | 0.693 |
| C17:1 *c*9 | HI | 0.05 | 0.006 | -0.02  0.02 | 0.006 | - | - | - | - | <0.001 | 0.002 | 0.133 | 0.902 |
|  | TM |  |  |  |  |  |  |  |  |  |  |  |  |
| C17:1 *t*10 |  | 0.03 | 0.007 | - | - | - | - | - | - | 0.001 | 0.216 | 0.776 | 0.515 |
|  |  |  |  |  |  |  |  |  |  |  |  |  |  |
| C18:1 *t*4 | HI | - | - | - | - | - | - | - | - | 0.092 | 0.135 | 0.729 | 0.686 |
|  | TM |  |  |  |  |  |  |  |  |  |  |  |  |
| C18:1 *t*5 | HI | 0.02 | 0.006 | -0.02 | 0.006 | - | - | - | - | 0.001 | 0.001 | 0.380 | 0.112 |
|  | TM |  |  | 0.02 |  |  |  |  |  |  |  |  |  |
| C18:1 *t*6-8 | HI | 0.34 | 0.096 | -0.29 | 0.096 | - | - | - | - | 0.001 | 0.005 | 0.116 | 0.137 |
|  | TM |  |  | 0.29 |  |  |  |  |  |  |  |  |  |
| C18:1 *t*9 | HI | - | - | -0.321 | 0.143 | - | - | 0.005 | 0.0026 | 0.202 | 0.034 | 0.451 | 0.048 |
|  | TM |  |  | 0.3209 |  |  |  | -0.005 |  |  |  |  |  |
| C18:1 *t*10-11 |  | - | - | - | - | - | - | - | - | 0.114 | 0.284 | 0.694 | 0.644 |
|  |  |  |  |  |  |  |  |  |  |  |  |  |  |
| C18:1 *t*12 |  | 0.27 | 0.078 | -0.21 | 0.078 | - | - | - | - | 0.002 | 0.012 | 0.125 | 0.259 |
|  |  |  |  | 0.21 |  |  |  |  |  |  |  |  |  |
| C18:1 *t*13+*t*14 |  | - | - | - | - | - | - | - | - | 0.484 | 0.645 | 0.997 | 0.913 |
|  |  |  |  |  |  |  |  |  |  |  |  |  |  |
| C18:1 *c*9 (+*c*10+*t*15) | HI | 12.43 | 0.900 | -6.46 | 0.900 | - | - | - | - | <0.001 | <0.001 | 0.661 | 0.440 |
|  | TM |  |  | 6.46 |  |  |  |  |  |  |  |  |  |
| C18:1 *c*11 | HI | 0.575 | 0.068 | -0.248 | 0.068 | - | - | - | - | <0.001 | 0.001 | 0.097 | 0.246 |
|  | TM |  |  | 0.248 |  |  |  |  |  |  |  |  |  |
| C18:1 *c*12 |  | 0.12 | 0.026 | - | - | - | - | - | - | <0.001 | 0.721 | 0.369 | 0.809 |
|  |  |  |  |  |  |  |  |  |  |  |  |  |  |
| C18:1 *c*13 |  | - | - | - | - | - | - | - | - | 0.498 | 0.224 | 0.236 | 0.164 |
|  |  |  |  |  |  |  |  |  |  |  |  |  |  |
| C18:1 *c*14 + *t*16 |  | 0.13 | 0.052 | - | - | - | - | - | - | 0.023 | 0.080 | 0.500 | 0.604 |
|  |  |  |  |  |  |  |  |  |  |  |  |  |  |
| C20:1 *c*11 |  | 0.03 | 0.007 | - | - | - | - | - | - | <0.001 | 0.648 | 0.657 | 0.469 |
|  |  |  |  |  |  |  |  |  |  |  |  |  |  |
| C18:2 *t*11*t*15 | HI | 0.057 | 0.015 | -0.046 | 0.015 | - | - | 0.001 | 0.0003 | 0.001 | 0.006 | 0.147 | 0.026 |
|  | TM |  |  | 0.046 |  |  |  | -0.001 |  |  |  |  |  |
| C18:2 *t*9*c*13 (+*t*8*c*12) |  | - | - | - | - | 0.0005 | 0.00022 | - | - | 0.917 | 0.729 | 0.032 | 0.219 |
|  |  |  |  |  |  |  |  |  |  |  |  |  |  |
| C18:2 *c*9*t*12+ C18:1 *c*16 |  | 0.38 | 0.071 | - | - | - | - | - | - | <0.001 | 0.065 | 0.489 | 0.501 |
|  |  |  |  |  |  |  |  |  |  |  |  |  |  |
| C18:2 *t*9*c*12 | HI | 0.10 | 0.017 | - | - | - | - | - | - | <0.001 | 0.075 | 0.226 | 0.218 |
|  | TM |  |  |  |  |  |  |  |  |  |  |  |  |
| C18:2 *t*11*c*15 | HI | 0.226 | 0.041 | -0.111 | 0.041 | - | - | 0.002 | 0.0007 | <0.001 | 0.012 | 0.118 | 0.011 |
|  | TM |  |  | 0.111 |  |  |  | -0.002 |  |  |  |  |  |
| C18:2 *c*9*c*15 |  | 0.02 | 0.011 | - | - | - | - | - | - | 0.044 | 0.677 | 0.748 | 0.563 |
|  |  |  |  |  |  |  |  |  |  |  |  |  |  |
| CLA *c*9*t*11 (+*t*7*c*9+*t*8*c*10) |  | - | - | - | - | - | - | - | - | 0.163 | 0.629 | 0.559 | 0.238 |
|  |  |  |  |  |  |  |  |  |  |  |  |  |  |
| CLA *t*11*c*13 (+*c*9*c*11) |  | 0.02 | 0.005 | - | - | - | - | - | - | 0.001 | 0.165 | 0.223 | 0.742 |
|  |  |  |  |  |  |  |  |  |  |  |  |  |  |
| CLA *t*10*c*12 |  | 0.03 | 0.012 | - | - | - | - | - | - | 0.020 | 0.793 | 0.979 | 0.291 |
|  |  |  |  |  |  |  |  |  |  |  |  |  |  |
| CLA *t*10*t*12 (+*t*11*t*13) |  | 0.13 | 0.050 | - | - | - | - | - | - | 0.017 | 0.745 | 0.840 | 0.836 |
|  |  |  |  |  |  |  |  |  |  |  |  |  |  |
| C18:3 n-3 |  | 0.83 | 0.182 | - | - | - | - | - | - | <0.001 | 0.329 | 0.276 | 0.710 |
|  |  |  |  |  |  |  |  |  |  |  |  |  |  |
| C20:4 n-6 |  | - | - | - | - | - | - | - | - | 0.786 | 0.869 | 0.621 | 0.569 |
|  |  |  |  |  |  |  |  |  |  |  |  |  |  |
| Total OCFA | HI | 1.39 | 0.111 | -0.52 | 0.111 | - | - | - | - | <0.001 | <0.001 | 0.535 | 0.553 |
|  | TM |  |  | 0.52 |  |  |  |  |  |  |  |  |  |
| Total BCFA |  | 5.60 | 1.215 | - | - | - | - | - | - | <0.001 | 0.080 | 0.431 | 0.480 |
|  |  |  |  |  |  |  |  |  |  |  |  |  |  |
| Total MUFA | HI | 19.76 | 2.331 | -10.12 | 2.331 | - | - | - | - | <0.001 | <0.001 | 0.891 | 0.735 |
|  | TM |  |  | 10.12 |  |  |  |  |  |  |  |  |  |
| Total C18:1 *c* | HI | 13.26 | 0.882 | -6.84 | 0.882 | - | - | - | - | <0.001 | <0.001 | 0.790 | 0.530 |
|  | TM |  |  | 6.84 |  |  |  |  |  |  |  |  |  |
| Total C18:1 *t* |  | - | - | - | - | - | - | - | - | 0.107 | 0.227 | 0.839 | 0.900 |
|  |  |  |  |  |  |  |  |  |  |  |  |  |  |
| Total n-3 FA |  | 1.14 | 0.212 | - | - | - | - | - | - | <0.001 | 0.118 | 0.174 | 0.733 |
|  |  |  |  |  |  |  |  |  |  |  |  |  |  |
| Total n-3 FA/Total n-6 FA |  | 1.41 | 0.370 | - | - | - | - | - | - | 0.001 | 0.594 | 0.835 | 0.624 |
|  |  |  |  |  |  |  |  |  |  |  |  |  |  |
| Total CLA |  | - | - | - | - | - | - | - | - | 0.082 | 0.640 | 0.652 | 0.331 |
|  |  |  |  |  |  |  |  |  |  |  |  |  |  |
| Total FA, g/kg DM |  | - | - | - | - | - | - | - | - | 0.537 | 0.745 | 0.415 | 0.869 |
|  |  |  |  |  |  |  |  |  |  |  |  |  |  |

*FA* Fatty acids, *HI* *Hermetia illucens*, *TM* *Tenebrio molitor*, *SEM* Standard error of the mean, *Temp* Drying temperature expressed as °C, *c* *cis*, *t* *trans*, *CLA* Conjugated linoleic acid, *OCFA* Odd-chain fatty acids, *BCFA* Branched-chain fatty acids, *MUFA* Monounsaturated fatty acids

## ^1^ The missing coefficients in the table (-) correspond to non significant effects, and thus are considered equal to zero
