# Supplementary material for: Low drying temperature has negligible impact but defatting increases in vitro rumen digestibility of insect meals, with minor changes on fatty acid biohydrogenation
Source: J Anim Sci Biotechnol. 2025 May 7;16:64. doi: 10.1186/s40104-025-01199-5 (PMC12056994; doi:10.1186/s40104-025-01199-5)
Supplement: Supplementary file 2 — Additional file 2: Table S2. Effect of ether extract content of insect meals on detailed fatty acid profile of rumen digesta, g/100 g total FA. [file 40104_2025_1199_MOESM2_ESM.docx]

## Table S2 Effect of ether extract content of insect meals on detailed fatty acid profile of rumen digesta, g/100 g total FA (unless otherwise stated)

| **Item^1^** | **Species** | **Intercept** | **Intercept**  **SEM** | **Species**  **coefficient** | **Species**  **SEM** | **EE**  **coefficient** | **EE**  **SEM** | **EE ×**  **Species**  **coefficient** | **EE ×**  **Species**  **SEM** | ***P-*value** | | | |
| --- | --- | --- | --- | --- | --- | --- | --- | --- | --- | --- | --- | --- | --- |
|  |  |  |  |  |  |  |  |  |  | **Intercept** | **Species** | **EE** | **EE ×**  **Species** |
| C10:0 | HI | 0.266 | 0.054 | 0.127 | 0.054 | - | - | 0.008 | 0.0030 | <0.001 | 0.026 | 0.092 | 0.012 |
|  | TM |  |  | -0.127 |  |  |  | -0.008 |  |  |  |  |  |
| C11:0 (+C10:1 *c*9) |  | 0.120 | 0.013 | - | - | - | - | - | - | <0.001 | 0.860 | 0.060 | 0.369 |
|  |  |  |  |  |  |  |  |  |  |  |  |  |  |
| C13:0 | HI | 0.017 | 0.002 | 0.005 | 0.002 | -0.0003 | 0.00013 | - | - | <0.001 | 0.038 | <0.001 | 0.577 |
|  | TM |  |  | -0.005 |  |  |  |  |  |  |  |  |  |
| C16:0 |  | 26.2 | 0.622 | - | - | - | - | - | - | <0.001 | 0.663 | 0.070 | 0.390 |
|  |  |  |  |  |  |  |  |  |  |  |  |  |  |
| C17:0 | HI | 0.59 | 0.080 | - | - | - | - | - | - | <0.001 | 0.095 | 0.136 | 0.315 |
|  | TM |  |  |  |  |  |  |  |  |  |  |  |  |
| C20:0 |  | 0.168 | 0.013 | - | - | -0.002 | 0.0007 | - | - | <0.001 | 0.597 | 0.003 | 0.840 |
|  |  |  |  |  |  |  |  |  |  |  |  |  |  |
| C21:0 (+CLA *t*9*c*11) | HI | 0.02 | 0.002 | 0.01 | 0.002 | - | - | - | - | <0.001 | 0.030 | 0.837 | 0.484 |
|  | TM |  |  | -0.01 |  |  |  |  |  |  |  |  |  |
| C22:0 |  | 0.052 | 0.008 | - | - | -0.001 | 0.0004 | - | - | <0.001 | 0.229 | 0.037 | 0.106 |
|  |  |  |  |  |  |  |  |  |  |  |  |  |  |
| C24:0 |  | - | - | - | - | 0.0001 | 0.00003 | - | - | 0.140 | 0.947 | 0.001 | 0.486 |
|  |  |  |  |  |  |  |  |  |  |  |  |  |  |
| C13 aiso | HI | 0.08 | 0.009 | - | - | - | - | - | - | <0.001 | 0.880 | 0.430 | 0.905 |
|  | TM |  |  |  |  |  |  |  |  |  |  |  |  |
| C18 iso | HI | 0.065 | 0.009 | -0.056 | 0.009 | 0.004 | 0.0005 | - | - | <0.001 | <0.001 | <0.001 | 0.142 |
|  | TM |  |  | 0.056 |  |  |  |  |  |  |  |  |  |
| C12:1 *c*9 | HI | 0.227 | 0.016 | -0.036 | 0.016 | -0.003 | 0.0009 | - | - | <0.001 | 0.028 | 0.001 | 0.610 |
|  | TM |  |  | 0.036 |  |  |  |  |  |  |  |  |  |
| C14:1 *t*9 | HI | 0.144 | 0.013 | - | - | - | - | -0.003 | 0.0007 | <0.001 | 0.809 | 0.466 | <0.001 |
|  | TM |  |  |  |  |  |  | 0.003 |  |  |  |  |  |
| C16:1 *c*7 |  | 0.609 | 0.059 | - | - | - | - | - | - | <0.001 | 0.242 | 0.309 | 0.054 |
|  |  |  |  |  |  |  |  |  |  |  |  |  |  |
| C17:1 *c*9 |  | 0.051 | 0.006 | - | - | - | - | - | - | <0.001 | 0.581 | 0.062 | 0.548 |
|  |  |  |  |  |  |  |  |  |  |  |  |  |  |
| C17:1 *t*10 | HI | 0.029 | 0.002 | -0.006 | 0.002 | - | - | - | - | <0.001 | 0.014 | 0.075 | 0.512 |
|  | TM |  |  | 0.006 |  |  |  |  |  |  |  |  |  |
| C18:1 *t*4 | HI | 0.01 | 0.002 | - | - | - | - | - | - | 0.005 | 0.945 | 0.448 | 0.804 |
|  | TM |  |  |  |  |  |  |  |  |  |  |  |  |
| C18:1 *t*5 | HI | 0.01 | 0.002 | - | - | - | - | - | - | <0.001 | 0.278 | 0.434 | 0.322 |
|  | TM |  |  |  |  |  |  |  |  |  |  |  |  |
| C18:1 *t*6-8 | HI | 0.063 | 0.009 | -0.030 | 0.009 | 0.002 | 0.0005 | 0.002 | 0.0005 | <0.001 | 0.001 | 0.004 | 0.003 |
|  | TM |  |  | 0.030 |  |  |  | -0.002 |  |  |  |  |  |
| C18:1 *t*9 | HI | 0.26 | 0.043 | 0.12 | 0.043 | - | - | - | - | <0.001 | 0.008 | 0.233 | 0.203 |
|  | TM |  |  | -0.12 |  |  |  |  |  |  |  |  |  |
| C18:1 *t*10-11 | HI | 0.477 | 0.172 | -0.496 | 0.172 | 0.075 | 0.0094 | 0.019 | 0.0094 | 0.010 | 0.007 | <0.001 | 0.050 |
|  | TM |  |  | 0.496 |  |  |  | -0.019 |  |  |  |  |  |
| C18:1 *t*12 | HI | 0.022 | 0.008 | -0.024 | 0.008 | 0.002 | 0.0005 | 0.0012 | 0.0005 | 0.012 | 0.008 | <0.001 | 0.012 |
|  | TM |  |  | 0.024 |  |  |  | -0.0012 |  |  |  |  |  |
| C18:1 *t*13+*t*14 | HI | 0.078 | 0.016 | -0.047 | 0.016 | 0.003 | 0.0009 | 0.003 | 0.0009 | <0.001 | 0.005 | 0.002 | 0.002 |
|  | TM |  |  | 0.047 |  |  |  | -0.003 |  |  |  |  |  |
| C18:1 *c*11 | HI | 0.763 | 0.038 | -0.139 | 0.038 | -0.006 | 0.0021 | 0.004 | 0.0021 | <0.001 | 0.001 | 0.006 | 0.046 |
|  | TM |  |  | 0.139 |  |  |  | -0.004 |  |  |  |  |  |
| C18:1 *c*12 | HI | 0.151 | 0.017 | -0.036 | 0.017 | - | - | - | - | <0.001 | 0.048 | 0.143 | 0.091 |
|  | TM |  |  | 0.036 |  |  |  |  |  |  |  |  |  |
| C18:1 c13 | HI | 0.08 | 0.026 | - | - | - | - | - | - | 0.004 | 0.167 | 0.187 | 0.404 |
|  | TM |  |  |  |  |  |  |  |  |  |  |  |  |
| C18:1 *c*14+*t*16 | HI | 0.050 | 0.007 | -0.020 | 0.007 | - | - | 0.001 | 0.0004 | <0.001 | 0.007 | 0.596 | 0.017 |
|  | TM |  |  | 0.020 |  |  |  | -0.001 |  |  |  |  |  |
| C20:1 *c*11 | HI | 0.05 | 0.012 | -0.03 | 0.012 | - | - | - | - | <0.001 | 0.044 | 0.427 | 0.242 |
|  | TM |  |  | 0.03 |  |  |  |  |  |  |  |  |  |
| C18:2 *t*11*t*15 |  | 0.062 | 0.010 | - | - | - | - | - | - | <0.001 | 0.209 | 0.234 | 0.058 |
|  |  |  |  |  |  |  |  |  |  |  |  |  |  |
| C18:2 *t*9*t*12 |  | -0.0066 | 0.002 | - | - | 0.0009 | 0.00013 | - | - | 0.009 | 0.545 | <0.001 | 0.590 |
|  |  |  |  |  |  |  |  |  |  |  |  |  |  |
| C18:2 *t*9*c*13 (+*t*8*c*12) | HI | 0.03 | 0.004 | - | - | - | - | - | - | <0.001 | 0.488 | 0.271 | 0.298 |
|  | TM |  |  |  |  |  |  |  |  |  |  |  |  |
| C18:2 *c*9*t*12+ C18:1 *c*16 |  | 0.661 | 0.029 | - | - | -0.009 | 0.0016 | - | - | <0.001 | 0.456 | <0.001 | 0.082 |
|  |  |  |  |  |  |  |  |  |  |  |  |  |  |
| C18:2 *t*9*c*12 | HI | 0.062 | 0.013 | 0.032 | 0.013 | 0.002 | 0.0007 | 0.003 | 0.0007 | <0.001 | 0.019 | 0.020 | 0.001 |
|  | TM |  |  | -0.031 |  |  |  | -0.003 |  |  |  |  |  |
| C18:2 *t*11*c*15 |  | 0.238 | 0.039 | - | - | -0.007 | 0.0021 | - | - | <0.001 | 0.054 | 0.004 | 0.176 |
|  |  |  |  |  |  |  |  |  |  |  |  |  |  |
| C18:2 *c*9*c*15 | HI | 0.035 | 0.004 | -0.013 | 0.004 | - | - | - | - | <0.001 | 0.003 | 0.056 | 0.624 |
|  | TM |  |  | 0.013 |  |  |  |  |  |  |  |  |  |
| CLA *t*11*c*13 (+*c*9*c*11) | HI | 0.01 | 0.002 | - | - | - | - | - | - | <0.001 | 0.510 | 0.172 | 0.192 |
|  | TM |  |  |  |  |  |  |  |  |  |  |  |  |
| CLA *t*10*c*12 |  | 0.0082 | 0.004 | - | - | 0.0010 | 0.00020 | - | - | 0.490 | 0.290 | <0.001 | 0.201 |
|  |  |  |  |  |  |  |  |  |  |  |  |  |  |
| CLA *t*10*t*12 (+*t*11*t*13) |  | 0.028 | 0.012 | - | - | 0.0033 | 0.0006 | - | - | 0.020 | 0.445 | <0.001 | 0.205 |
|  |  |  |  |  |  |  |  |  |  |  |  |  |  |
| C20:2 n-6 |  | - | - | - | - | 0.0004 | 0.00010 | - | - | 0.061 | 0.445 | <0.001 | 0.222 |
|  |  |  |  |  |  |  |  |  |  |  |  |  |  |
| C20:4 n-6 | HI | - | - | - | - | - | - | 0.0006 | 0.00024 | 0.053 | 0.706 | 0.115 | 0.019 |
|  | TM |  |  |  |  |  |  | -0.0006 |  |  |  |  |  |
| Total C18:1 *c* | HI | 14.33 | 0.732 | -1.72 | 0.732 | - | - | -0.22 | 0.040 | <0.001 | 0.026 | 0.068 | <0.001 |
|  | TM |  |  | 1.72 |  |  |  | 0.22 |  |  |  |  |  |
| Total C18:1 *t* | HI | 0.91 | 0.196 | -0.48 | 0.196 | 0.08 | 0.011 | 0.022 | 0.011 | <0.001 | 0.021 | <0.001 | 0.045 |
|  | TM |  |  | 0.48 |  |  |  | -0.022 |  |  |  |  |  |
| Total CLA | HI | - | - | - | - | 0.016 | 0.0021 | - | - | 0.878 | 0.348 | <0.001 | 0.131 |
|  | TM |  |  |  |  |  |  |  |  |  |  |  |  |

## *FA* Fatty acids, *HI* *Hermetia illucens*, *TM* *Tenebrio molitor*, *SEM* Standard error of the mean, *EE* Ether extract expressed as g/100g DM, *c* *cis*, *t* *trans*, *CLA* Conjugated linoleic acids

## ^1^ The missing coefficients in the table (-) correspond to non significant effects, and thus are considered equal to zero
